# Prenatal Biochemical and Ultrasound Markers in COVID-19 Pregnant Patients: A Prospective Case-Control Study

**DOI:** 10.3390/diagnostics11030398

**Published:** 2021-02-26

**Authors:** Stefano Cosma, Andrea Roberto Carosso, Fulvio Borella, Jessica Cusato, Marialuisa Bovetti, Federica Bevilacqua, Marco Carosso, Fiammetta Gervasoni, Andrea Sciarrone, Luca Marozio, Alberto Revelli, Alessandro Rolfo, Claudia Filippini, Valeria Ghisetti, Giovanni Di Perri, Chiara Benedetto

**Affiliations:** 1 Gynecology and Obstetrics 1, Department of Surgical Sciences, City of Health and Science, University of Turin, 10126 Turin, Italy; andrea88.carosso@gmail.com (A.R.C.); fulvio.borella87@gmail.com (F.B.); marialuisa.bovetti@unito.it (M.B.); fede.bevi23@gmail.com (F.B.); marco.carosso94@gmail.com (M.C.); fiammetta.gervasoni@gmail.com (F.G.); luca.marozio@unito.it (L.M.); alberto.revelli@unito.it (A.R.); chiara.benedetto@unito.it (C.B.); 2 Laboratory of Clinical Pharmacology and Pharmacogenetics, Amedeo di Savoia Hospital, Department of Medical Sciences, University of Turin, 10126 Turin, Italy; jessica.cusato@unito.it; 3 Obstetrics-Gynecological Ultrasound and Prenatal Diagnosis Unit, Department of Obstetrics and Gynecology, City of Health and Science, 10126 Turin, Italy; asciarrone@cittadellasalute.to.it; 4 Department of Surgical Sciences, City of Health and Science, University of Turin, 10126 Turin, Italy; alessandro.rolfo@unito.it (A.R.); claudia.filippini@unito.it (C.F.); 5 Laboratory of Microbiology and Virology, Amedeo di Savoia Hospital, ASL ‘Città di Torino’, 10126 Turin, Italy; valeria.ghisetti@gmail.com; 6 Unit of Infectious Diseases, Amedeo di Savoia Hospital, Department of Medical Sciences, University of Turin, 10126 Turin, Italy; giovanni.diperri@unito.it

**Keywords:** COVID-19, fetal congenital anomalies, fetus, first trimester, nuchal translucency, prenatal screening test, SARS-CoV-2, vertical transmission

## Abstract

This prospective observational study aimed to evaluate whether women with SARS-CoV-2 infection during the first trimester of pregnancy are at higher risk of noninvasive prenatal screening test alterations and/or of congenital fetal anomalies at the second-trimester fetal anatomy scan. Maternal symptoms were secondly investigated. The study was carried out on 12-week pregnant women admitted for noninvasive prenatal testing (16 April and 22 June 2020). The cohort had seromolecular tests for SARS-CoV-2, after which they were divided into a positive case group and a negative control group. Both groups had 20-week ultrasound screening. Seventeen out of the 164 women tested positive for SARS-CoV-2 (10.3%). There were no significant differences in mean nuchal translucency thickness or biochemical markers (pregnancy-associated plasma protein A, alpha-fetoprotein, human chorionic gonadotropin, unconjugated estriol) between cases and controls (*p* = 0.77, 0.63, 0.30, 0.40, 0.28) or in the fetal incidence of structural anomalies at the second-trimester fetal anatomy scan (*p* = 0.21). No pneumonia or hospital admission due to COVID-19-related symptoms were observed. Asymptomatic or mildly symptomatic SARS-CoV-2 infection during the first trimester of pregnancy did not predispose affected women to more fetal anomalies than unaffected women. COVID-19 had a favorable maternal course at the beginning of pregnancy in our healthy cohort.

## 1. Introduction

Coronavirus disease-19 (COVID-19), the infectious disease caused by the newly discovered coronavirus (SARS-CoV-2) was first reported in Wuhan (China) in December 2019 and officially declared as a pandemic by the World Health Organization (WHO) on 11 March 2020 [1]. Viral vertical transmission from mother to fetus has been a major problem with emergent viruses such as human Immunodeficiency virus, Ebola virus, and Zika virus more recently [2,3,4]. Overall, the transmission rates for viral pathogens range from as low as 0.2–0.4% for Cytomegalovirus and Varicella-Zoster virus to as high as 17–33% for Parvovirus B19, which is also associated with increased nuchal translucency (NT) thickness [5].

In a prospective pilot study, Coronaviruses (229E, OC-43, NL-63, HKU1) were detected in seven mother-infant dyads out of 159 (4.4%) [6]. There were no confirmed cases of intrauterine maternal-fetal transmission of SARS-CoV and MERS-CoV identified in pregnant women infected during the 2002–2003 and 2012 epidemics, although maternal death and obstetrical complications occurred as a result of the infection [7].

As of this writing, we have no information about the potential effects of SARS-CoV-2 infection on early embryogenesis and organogenesis. Early congenital transmission of SARS-CoV-2 in humans is plausible by analogy with animal models of coronavirus disease [8]. In addition, there is mounting evidence for a risk of vertical transmission during the third trimester, from 3.2% based on infant nasopharyngeal (NF) swabs to 7.7% based on placental sample analysis [9,10,11,12,13].

The primary aim of this study was to determine whether SARS-CoV-2 positive women during the first trimester of pregnancy are at higher risk of noninvasive prenatal screening test alterations and/or of congenital anomalies at the second-trimester fetal anatomic survey (18–22 weeks) compared to a cohort of healthy pregnant women. The secondary aim was to evaluate maternal symptoms.

## 2. Materials and Methods

Consecutive 12-week pregnant patients attending our institution for noninvasive prenatal diagnosis or admitted to the care units for obstetric or COVID-19-related symptoms, between 16 April and 22 June 2020 were invited to participate in the study.

NF swabs were taken for reverse transcriptase-polymerase chain reaction (RT-PCR) assay for detection of SARS-CoV-2; blood samples taken for prenatal screening were used for detection of antibodies against SARS-CoV-2. Semi-quantitative detection of IgG/IgM non-neutralizing antibodies (nNAbs) was performed by an automated (AFIAS™ COVID-19, Boditech Med Inc, Gang-won-do, Korea) lateral flow immunochromatographic assay; semi-quantitative detection of IgG neutralizing antibodies (NAbs) was performed by a chemiluminescent immunoassay (Liaison^®^ SARS-CoV-2 S1/S2 IgG, DiaSorin, Saluggia, Italy). Both serologic assays had Emergency Use Authorization.

**Figure 1:**
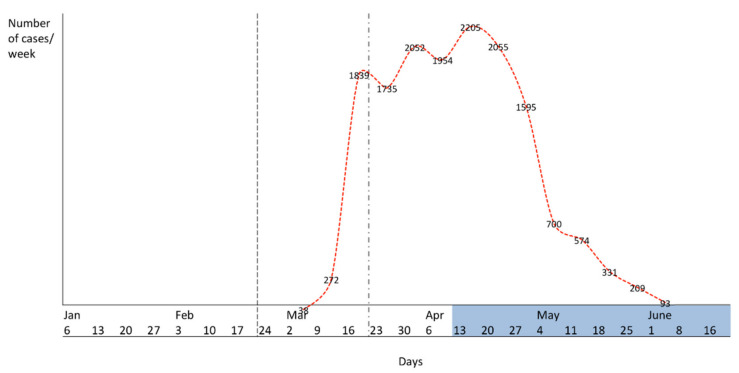
Last menstruation criterion for inclusion and recruitment period. Dotted black line: first reported case of COVID-19 in Piedmont, Italy; dash-dotted black line: date representing the last menstruation upper limit of inclusion; dotted red line: COVID-19 outbreak cases in Turin, weekly case increase; highlighted blue: recruitment period

Only women with last menstruation at most one month later than the date of the first reported case of COVID-19 infection in Piedmont (22 February 2020) were considered eligible for inclusion in the study, so as to exclude the possibility of COVID-19 seroconversion before pregnancy (Figure 1). The duration of SARS-CoV-2 virus shedding varies up to 6–8 weeks in upper respiratory tract samples and 5 weeks in feces [14]. This strict recruitment criterion allowed us to define seropositivity as a seroconversion that had occurred during pregnancy. Exclusion criteria were delivery scheduled at another hospital, inability to give informed consent, and aged less than 18 years old.

Positive patients were defined as those who tested positive by NAbs or nNAbs or NF swab.

The virological test results at 12 weeks of pregnancy were collected and the cohort was divided into two groups: the first trimester SARS-CoV-2-positive patients, both asymptomatic and symptomatic (case group) and the SARS-CoV-2-negative women (control group). Patients with COVID-19 were managed according to a standard regional protocol. The entire cohort was seromolecular tested a second time at 16 weeks of pregnancy and a third time at 21 weeks. The patients in the control group who tested positive at the second or the third step were excluded from the study, in order to select only first trimester seroconversions.

Both groups were followed longitudinally throughout pregnancy by the study team. Demographics, screening test results, sonographic data, COVID-19-related symptoms and obstetric outcomes were collected. A telephone number was provided to ensure that the study team could be contacted in case hospital admission became necessary.

### 2.1. Prenatal Testing

While screening strategies may vary, the Prenatal Diagnosis Center at our hospital offers alongside the first trimester fetal aneuploidy combined test, an integrated first and second trimester test that provides a single revised risk assessment for fetal aneuploidy or open neural tube defect based on NT measurement plus pregnancy-associated plasma protein A (PAPP-A) in the first trimester (11–12 weeks) and alpha-fetoprotein (AFP), human chorionic gonadotropin (hCG), unconjugated estriol (uE3) in the second trimester (15–16 weeks) [15].

When an invasive prenatal test (e.g., amniocentesis) was performed on a SARS-CoV-2-positive patient, a sample of the amniotic fluid was also collected for SARS-CoV-2 detection. Comparative genomic hybridization with molecular cytogenetic analysis for the detection and mapping of chromosomal gains and losses was used.

The second-trimester fetal anatomic survey on COVID-19 positive patients was carried out in the Sant Anna Prenatal Diagnosis Center; cardiac activity, fetal size, basic fetal anatomy and wellbeing, and placental appearance and location were assessed according to the according to the International Society of Ultrasound in Obstetrics and Gynecology (ISUOG) guidelines [16].

### 2.2. Diagnostic Assays

Viral RNA was extracted from the swab using a MagNA Pure Compact nucleic acid isolation kit (Roche, Mannheim, Germany) and analyzed by a RT-PCR assay (CFX-96, Bio-Rad, Milan, Italy) with the Liferiver Novel Coronavirus 2019-nCov real-time RT-PCR kit protocol, targeting genes N, E, and ORF1ab (Liferiver Bio-Tech, San Diego, CA, USA). Fluorescent lateral flow assays were performed to detect IgG/IgM nNAb against the nucleocapsid (N) viral proteins. The AFIAS™ COVID-19 gives semi-quantitative results expressed as a cut-off index (COI) where a COI of >1.1 indicates a positive result. Chemiluminescent immunoassay technology was used for semi-quantitative determination of anti-S1 and anti-S2 specific IgG neutralizing antibodies to SARS-CoV-2 (Liaison^®^ SARS-CoV-2 S1/S2 IgG, Diasorin): the antibody concentration is expressed as arbitrary units (AU/mL) and grades the results as positive when ≥15 AU/mL.

### 2.3. Statistical Analysis

Pearson’s chi-square test or Fisher’s exact test as appropriate was used for categorical variables, and Student’s *t*-test for normally distributed variables or Wilcoxon matched-pairs signed-rank test otherwise. For all comparisons, differences were considered statistically significant if *p* < 0.05. Statistical analyses were performed using SAS software version 9.4 for Windows (SAS Institute, Carey, NC, USA).

The effect of COVID-19 infection in the first trimester of pregnancy on NT thickness, PAPP-A, hCG, AFP, uE3 was assessed using a generalized linear multivariable model taking maternal age and gestational age into account. A *p* < 0.05 was considered statistically significant. We did not report missing data for each variable of interest.

Sample size calculation was not possible because the effect size of COVID-19 on the outcomes evaluated in this study is unknown and cannot be estimated on the basis of previous publications. Moreover, disease prevalence was unpredictable as it is dependent on the epidemic curve at enrollment; finally, the size of cohort was strictly limited by the inclusion criteria as further recruitment beyond 22 June would have precluded the eligibility criterion for the last menstruation.

## 3. Results

**Figure 2:**
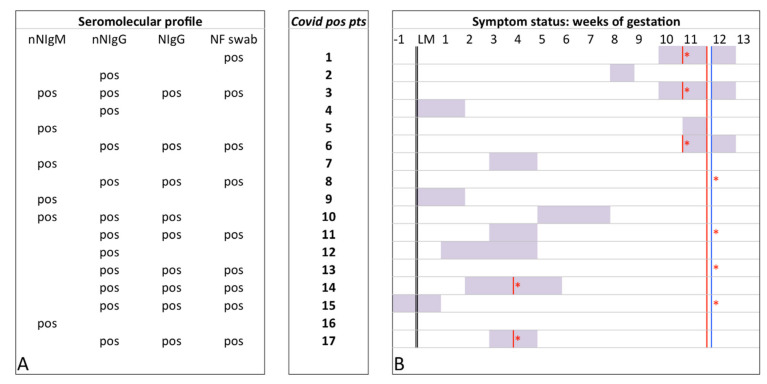
COVID-19 affected patients: seromolecular profile (**A**) and symptom status (**B**). Blue vertical line, serological sampling; NIgG, neutralizing IgG; nNIgG, non-neutralizing IgG; nNIgM, non-neutralizing IgM; NF, nasopharyngeal; pos, positive; pts, patients; purple box, time window of symptoms; red vertical line, molecular sampling; red *, molecular positive sampling.

A total of 164 out of 192 women in their first trimester of pregnancy, attending our institute during the recruitment period, were included in the study. The patient attendance rate was 85.4% (164/192). Seventeen of the 164 women tested for anti-SARS-CoV-2 IgG and IgM antibodies at 12 weeks were seropositive or had a positive NF swab test for SARS-CoV-2, yielding an overall COVID-19 cumulative incidence of 10.4% in the first trimester. Sixteen (16/164, 9.7%) tested positive for nNAbs: 10/16 (62.5%), 4/16 (25%), and 2/16 (12.5%) were positive for SARS-CoV-2 nNIgG, IgM, or both IgG and IgM, respectively; one had a positive NF swab without antibody response. Nine of the 16 (56.2%) who tested positive for nNAbs (IgM and/or IgG) were also positive for NAbs (IgG). The NF swab tested positive in 9/164 (5.5%) (Figure 2A).

**Figure 3:**
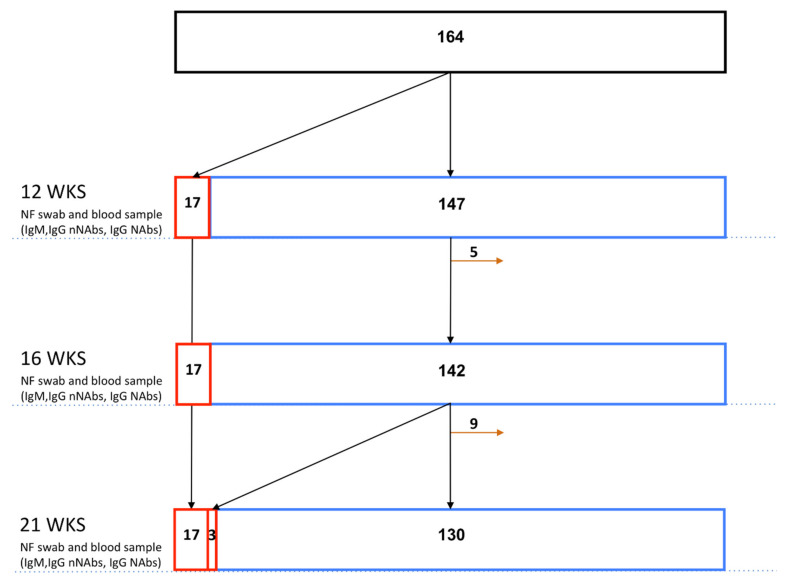
Study flow diagram: case ascertainment and control selection. nNAbs, non-neutralizing antibodies; NAbs, neutralizing antibodies; NF, nasopharyngeal; orange arrow, patients excluded; WKS, weeks of gestation.

The data from 17 women in the case group and 130 in the control group were entered into the final analysis, whilst those with seroconversion in the second trimester and patients lost to follow-up were excluded; one spontaneous abortion in the control group was also not considered for the analysis as it happened before the prenatal testing (Figure 3).

Table 1 presents the patients’ baseline characteristics; there were no statistically significant differences in demographics or comorbidities between the two groups.

### 3.1. Fetal Outcomes

There were no statistically significant differences in mean NT thickness, PAPP-A, AFP, hCG or uE3 levels between the positive women and those with negative levels of SARS-CoV-2 antibodies. Also, after accounting for maternal age and gestational age, positive antibodies did not affect NT thickness, PAPP-A, AFP, hCG or uE3 levels (Table 2).

Prenatal diagnosis was performed in 16/17 COVID-19 positive patients and one patient declined. There were no statistically significant differences in positive screening tests between the two groups (Table 2); additional investigations (1 amniocentesis and 2 cell-free fetal DNA in maternal blood -case group-; 3 amniocentesis and 26 cell-free fetal DNA in maternal blood -control group-) yielded negative results. The positive screening test in the case group showed low hCG (7.46 IU/L, 0.24 MoM) and PAPP-A (521.00 IU/L, 0.14 MoM) levels in a 35-year-old COVID-19-positive patient. Subsequent analysis of cell-free fetal DNA in maternal blood and 20-week scan yielded negative results. She referred mild fever for 3 days, followed by ageusia for 3 weeks and her blood profile showed COVID-19 IgM-IgG nNAbs (1.04–21.04 COI) and IgG NAbs (52.70 IU/L) (3; Figure 2B).

There were no statistically significant differences in anatomy scan findings between the two groups (Table 3). A fetal malformation in the case group and an orofacial cleft in the control group were noted, without reaching statistical difference (*p* = 0.21). A 35-year-old COVID-19-positive patient presenting at the 20-week screen scan with fetal right ventricular dominance, double superior vena cava, and severe atrial septal defect underwent invasive prenatal testing (amniocentesis). The amniotic fluid sample tested negative for SARS-CoV-2 and aneuploidies were excluded. The patient reported fever, cough, and sore throat for a few days at 8 weeks of gestation (2; Figure 2B). At inclusion into the study (12 weeks), she was asymptomatic, and her blood profile showed COVID-19 IgG nNAbs (19.28 COI). Integrated screening test yielded negative results. Another COVID-19-positive patient had fetal bladder dilation at the 12-week ultrasound scan, which was no longer present on subsequent ultrasound scans, nor were any other urinary tract anomalies.

At time of writing, 132/147 women included in the final analysis were still pregnant (range of gestational age, 28 to 40 weeks). No spontaneous abortion in the case group was recorded.

### 3.2. Maternal Outcomes

The number of symptoms suggestive of COVID-19 prior to enrolment into the study was higher in the women who tested positive (case group) than in those who did not (control group) (14/17, 82.3% vs. 22/130, 16.9%; *p* < 0.001).

No pneumonia or hospital admission due to COVID-19-related symptoms were recorded.

In the case group, 5/17 (29.4%) (1, 3, 6, 14, 17; Figure 2B) patients who tested positive at RT-PCR for SARS-CoV-2 before 12 weeks of pregnancy were self-reported as being symptomatic, including 3 who attended prenatal screening tests through the dedicated path for SARS-CoV-2 positive patients. Three out of 17 (17.6%) (8, 13, 16; Figure 2B) were asymptomatic; 9/17 (52.9%) had self-misrecognized symptoms and reported symptoms only at history taking (2, 4, 5, 7, 9, 10, 11, 12, 15; Figure 2B). Reported symptoms included fever (7/12, 58.3%), anosmia and ageusia (6/12, 50%), cough (5/12, 41.7%), arthralgia (4/12, 33.3%), diarrhea (3/12, 25%) and dyspnea (2/12, 16.7%). The only comorbid conditions in the case group, linked to poor outcomes in SARS-CoV-2 infection were: 2 overweight patients with a BMI >25 and one with controlled type 1 diabetes.

Nine out 12 patients who seroconverted to IgG antibodies, expressed IgG NAbs (75%), regardless of the presence of symptoms. The mean antibody titer at admission was 20.52 COI and 1.18 COI for anti-SARS-CoV-2 IgG and IgM nNAbs and 43.72 AU/mL for anti-SARS-CoV-2 IgG NAbs.

## 4. Discussion

### 4.1. Key Results

Previous or ongoing COVID-19 was detected in about 1/10 pregnant women attending the noninvasive prenatal services of our hospital [18]. There were no significantly different unfavorable prenatal biochemical or ultrasound markers of fetal anomalies in women with COVID-19 in the first trimester compared to a cohort of SARS-CoV-2 negative pregnant women matched for gestational age; a single case of CHD (congenital heart disease) was observed at the 20-week ultrasound scan. However, after excluding congenital chromosomal anomalies no association with COVID-19 was clearly demonstrated, as the RT-PCR for SARS-CoV-2 in the amniotic fluid sample was negative. We also reported one patient with abnormal low values of hCG and PAPP-A. However, these two patients with impaired prenatal markers of fetal anomalies do not share common clinical features, either with regard to symptoms or antibody response. Lastly, the incidence of maternal symptoms in our case series was negligible and no symptomatic patient required hospital admission.

### 4.2. Strengths and Limitations

The main strength and limitation of our prospective study were both determined by the same strict inclusion criterion that did not allow recruitment of patients later than one month from the date of the first reported case of COVID-19 in Piedmont. Whilst on the one hand this guaranteed a selection of a case group with an infection that most certainly occurred in the first trimester, on the other it limited the sample size and increased the probability of committing a type II error. However, the inclusion of a control group was essential to correctly measure the impact of SARS-CoV-2 infection on pregnancy and reduce the risk of overestimation.

We included nNIgM-positive patients (5, 7, 9, 16; Figure 2A): limited and discordant evidence for immune response kinetics following SARS-CoV-2 infection did not allow, especially considering the symptoms these patients reported, to exclude them from the case group. The immunoprofile at term of pregnancy will help to distinguish a false positive result for cross-reactivity from a rare isolated IgM response or from a late IgG seroconversion [19,20].

Lastly, our case group was relatively healthy, without underlying uncontrolled medical conditions associated with worsening the severity of the coronavirus infection; therefore, no conclusions as to the impact of COVID-19 in pregnant patients with comorbidities during the first trimester can be drawn. Although the study aimed also at the inclusion of hospitalized patients, a selection bias cannot be excluded as the recruitment method may have led mainly to the inclusion of asymptomatic women, as SARS-CoV-2 positive patients with serious symptoms would not have attended the scheduled hospital appointment.

### 4.3. Interpretation

In light of our results, the interpretation of the fetal favorable outcomes might follow two pathophysiological considerations: first, low co-expression of the angiotensin-converting enzyme 2 (ACE2) and transmembrane serine protease 2 (TMPRSS2) SARS-CoV-2 receptors at maternal-fetal interface and in fetal target organs during early pregnancy; second, asymptomatic or mild clinical presentation results in a low probability of viraemia.

SARS-COV-2 requires, besides the ACE2 receptor, the cellular protease TMPRSS2 to cleave the viral spike protein and facilitate the fusion of viral and cellular target membranes. Single-cell transcriptomic data on single-nuclear RNA-sequencing from first-trimester placentas demonstrated that co-expression of ACE2 and TMPRSS2 is negligible at this stage (<1/10,000 cells) [21]. These findings could also explain the recent evidence showing no increase in early pregnancy loss in patients with SARS-CoV-2 infection [22,23]. Conversely, the viral receptors used by Cytomegalovirus, Zika virus and other viruses are highly expressed by human placental tissues [24].

Moreover, viral infection of the cells at the maternal-fetal interface is a necessary but not sufficient condition to induce direct fetal teratogenic effects. Organs without ACE2 expression are not the target of virus-related insult. There is conflicting evidence for the expression of ACE2 and TMPRSS2 receptors at the fetal organs. The recently reported absence of ACE2 expression by immunochemistry, in fetal brain and heart tissue appears reassuring for the risk of congenital organ-specific anomalies, although only 5 cases were analyzed [25].

In contrast, previous single-cell RNA sequencing data revealed that ACE2 is highly expressed in the fetal heart, liver and lung. Interestingly, only low-level ACE2 expression in the early placenta (8 weeks) was observed, whilst the expression significantly increased at later stages of pregnancy (24 weeks), as was for TMPRSS2 [26].

Finally, our findings show that COVID-19 during the first trimester of pregnancy is often asymptomatic. As viraemia is usually only detectable in around 1% of symptomatic adults, it might even be less in asymptomatic pregnant women, which makes blood-borne transmission unlikely at a very early gestation age [27].

Similar to the course of the disease in non-pregnant adults, 80–90% of infections were not severe. The percentage of patients without or with self-misrecognized symptoms (70.6%) was in line with current data on the third trimester of pregnancy, suggesting that asymptomatic women may account for 72% of SARS-CoV-2 infections [28]. The low incidence of severe manifestations during the first trimester could be explained by the minimal alteration in respiratory dynamics during this phase of pregnancy. Our cohort members most likely did not realize that their symptoms were suggestive of COVID-19 (52.9%), as all but five had never been tested for SARS-CoV-2 before.

The prevalence of symptoms (self-reported or misrecognized) was five-fold higher in those testing positive (82.3%) than in those testing negative (16.9%), suggesting that pregnant women in epidemic areas with symptoms should consult with their obstetrician about whether to be tested for COVID-19. Self-reported symptoms, like ageusia, anosmia, together with fever or cough, can help to identify 87.5% of symptomatic COVID-19 cases [29].

### 4.4. Generalisability

In conclusion, although no evidence for unfavorable prenatal biochemical and ultrasound markers of fetal anomalies were observed in our COVID-19-positive patients, the fetal cardiac malformation poses a dilemma as a history of maternal viral infection, including various types of viruses, in early pregnancy may be associated with an increased risk of CHD in the offspring [30]. The involved patient did not seroconvert to NAbs; we can speculate that the presence of nNAbs alone or with suboptimal neutralizing activity may bear risks. For instance, antibodies that fail to inhibit its binding with the viral receptor can interact with the Fc gamma receptors to enter target cells, potentially causing antibody-dependent enhancement and increased levels of inflammatory cytokines [31].

Although our data cannot be generalized, as they come from limited observations, they do emphasize the need to plan further investigation with large, multicenter observational studies to asses any etiopathogenetic link between SARS-CoV-2 infection in early pregnancy and fetal anomalies.

Such findings are pivotal to guide correct pre-conception counseling on the risks of pregnancy in COVID-19 epidemic areas and to plan follow-up during pregnancy with the aim of reducing fetal and maternal morbidity.

## Figures and Tables

**Table 1:** Baseline characteristics, clinical findings in COVID-19-positive and -negative patients.

Clinical Findings		COVID-19 Positive *n* = 17	COVID-19 Negative *n* = 130	*p*
		*n* (%) or mean ±SD	*n* (%) or mean ±SD	
Age (years)		32.1 ± 4.0	33.9 ± 4.3	0.16
BMI prior to pregnancy, (kg/m^2^)		22.3 ± 3.2	22.7 ± 4.2	0.88
Parity				0.68
	0	9 (52.9)	82 (63.8)	
	1	7 (41.2)	37 (28.5)	
	2	1 (5.9)	9 (6.9)	
	3	0 (0)	2 (1.5)	
Previous				0.28
Abortions	0	14 (82.3)	95 (73.0)	
	1	1 (5.9)	25 (19.2)	
	2	1 (5.9)	8 (6.1)	
	3	1 (5.9)	2 (1.5)	
ART therapy		2 (11.8)	9 (6.9)	0.47
Smoking history		1 (5.9)	9 (6.9)	0.87
Thyroid disease		1 (5.9)	14 (10.8)	0.53
Autoimmune diseases		0 (0)	4 (3.1)	0.46
Thrombophilia		1 (5.9)	5 (3.8)	0.68
Uncontrolled DM		0	0	
Uterine anomalies		0 (0)	8 (6.1)	0.29

ART, assisted reproductive technique; BMI, body- mass index; DM, diabetes mellitus; SD, standard deviation.

**Table 2:** Noninvasive prenatal screening test findings in COVID-19-positive and -negative patients.

	COVID-19 Positive *n* = 17	COVID-19 Negative *n* = 130	*p*	*p* *
GA at time of screening test, days ±SD	87.2 ± 2.3	86.3 ± 1.8	0.49	/
Integrated test, n (%)	15 (93.7)	114 (87.6)	/	/
Combined test, n (%)	1 (6.2)	16 (12.3)	/	/
Positive screening test, n (%)	1 (6.2)	4 (3.0)	0.46	/
Risk for Down’s syndrome, ratio ±SD	1/43,493.1 ± 1/42,876.8	1/39,880.1 ± 1/39,357.5	0.88	/
Risk for neural tube defects, ratio ±SD	1/5178.6 ± 1696.2	1/4600.0 ± 1649.4	0.20	/
NT thickness, mm ±SD	1.4 ± 0.2	1.4 ± 0.3	0.77	0.89
NT thickness, MoM ±SD	1.0 ± 0.1	1.1 ± 0.3	0.12	0.11
PAPP-A, IU/L ±SD	3216.6 ± 1514.3	3256.0 ± 2194.8	0.63	0.95
PAPP-A, MoM ±SD	1.3 ± 0.9	1.2 ± 0.7	0.55	0.62
AFP, IU/L ±SD	27.8 ± 12.0	28.5 ± 8.4	0.30	0.81
AFP, MoM ±SD	1.0 ± 0.4	1.0 ± 0.2	0.15	0.41
hCG, IU/L ±SD	32.3 ± 17.1	34.8 ± 16.2	0.40	0.50
hCG, MoM ±SD	1.0 ± 0.5	1.1 ± 0.5	0.59	0.63
uE3, IU/L ±SD	0.9 ± 0.2	0.9 ± 0.2	0.28	0.13
uE3, MoM ±SD	1.1 ± 0.2	1.1 ± 0.2	0.58	0.40

AFP, alpha-fetoprotein: GA, gestational age, hCG, human chorionic gonadotropin; IU/L, international units per liter; MoM, multiples of the median; NT, nuchal translucency; uE3, unconjugated estriol; SD, standard deviation; PAPP-A, pregnancy-associated plasma protein; * multivariate linear model analysis.

**Table 3:** Second-trimester fetal anatomy scan findings in COVID-19-positive and -negative patients.

	COVID-19 Positive *n* = 17	COVID-19 Negative *n* = 130	*p*
	*n* (%)	*n* (%)	
Estimated fetal HC Z-score ≥ 1.645	2 (11.7)	19 (14.6)	0.75
Estimated fetal AC Z-score ≥ 1.645	2 (11.7)	11 (8.4)	0.65
Estimated fetal FL Z-score ≥ 1.645	3 (17.6)	14 (10.7)	0.40
Estimated fetal HC Z-score ≤ −1.28	1 (5.8)	4 (3.0)	0.54
Estimated fetal AC Z-score ≤ −1.28	1 (5.8)	4 (3.0)	0.54
Estimated fetal FL Z-score ≤ −1.28	0	1 (0.7)	0.71
Fetal structural anomalies	1 (5.8)	1 (0.7)	0.21

AC, abdominal circumference; HC, head circumference; FL: femur length; Z-scores (standard deviations) were estimated by INTERGROWTH-21st standards [17].

## Data Availability

The data presented in this study are available on request from the corresponding author.

## References

[1] Coronavirus Disease (COVID-19)—Events as They Happen. https://www.who.int/emergencies/diseases/novel-coronavirus-2019/events-as-they-happen.

[2] Newell M.L. (2000). Vertical transmission of HIV-1 infection. Trans. R. Soc. Trop. Med. Hyg..

[3] Bebell L.M., Oduyebo T., Riley L.E. (2017). Ebola virus disease and pregnancy: A review of the current knowledge of Ebola virus pathogenesis, maternal, and neonatal outcomes. Birth Defects Res..

[4] Alvarado M.G., Schwartz D.A. (2017). Zika Virus Infection in Pregnancy, Microcephaly, and Maternal and Fetal Health: What We Think, What We Know, and What We Think We Know. Arch. Pathol. Lab. Med..

[5] Grubman O., Hussain F.N., Nelson Z., Brustman L. (2019). Maternal Parvovirus B19 Infection Causing First-Trimester Increased Nuchal Translucency and Fetal Hydrops. Case Rep. Obstet. Gynecol..

[6] Gagneur A., Dirson E., Audebert S., Vallet S., Legrand-Quillien M.C., Laurent Y., Collet M., Sizun J., Oger E., Payan C. (2008). Materno-fetal transmission of human coronaviruses: A prospective pilot study. Eur. J. Clin. Microbiol. Infect Dis..

[7] Rasmussen S.A., Smulian J.C., Lednicky J.A., Wen T.S., Jamieson D.J. (2020). Coronavirus Disease 2019 (COVID-19) and Pregnancy: What obstetricians need to know. Am. J. Obstet. Gynecol..

[8] Singh A., Singh R.S., Sarma P., Batra G., Joshi R., Kaur H., Sharma A.R., Prakash A., Medhi B. (2020). A Comprehensive Review of Animal Models for Coronaviruses: SARS-CoV-2, SARS-CoV, and MERS-CoV. Virol Sin..

[9] Carosso A., Cosma S., Serafini P., Benedetto C., Mahmood T. (2020). How to reduce the potential risk of vertical transmission of SARS-CoV-2 during vaginal delivery?. Eur. J. Obstet. Gynecol. Reprod. Biol..

[10] Carosso A., Cosma S., Benedetto C. (2020). Vaginal delivery in COVID-19 pregnant women: Anorectum as a potential alternative route of SARS-CoV-2 transmission. Am. J. Obstet. Gynecol..

[11] Carosso A., Cosma S., Borella F., Marozio L., Coscia A., Ghisetti V., Di Perri G., Benedetto C. (2020). Pre-labor anorectal swab for SARS-CoV-2 in COVID-19 pregnant patients: Is it time to think about it?. Eur. J. Obstet. Gynecol. Reprod. Biol..

[12] Di Mascio D., WAPM (The World Association of Perinatal Medicine) working group on COVID-19 (2020). Maternal and Perinatal Outcomes of Pregnant Women with SARS-COV-2 infection. Ultrasound Obstet. Gynecol..

[13] Kotlyar A.M., Grechukhina O., Chen A., Popkhadze S., Grimshaw A., Tal O., Taylor H.S., Tal R. (2020). Vertical transmission of coronavirus disease 2019: A systematic review and meta-analysis. Am. J. Obstet. Gynecol..

[14] Sun J., Tang X., Bai R., Liang C., Zeng L., Lin H., Yuan R., Zhou P., Huang X., Xiong Q. (2020). The kinetics of viral load and antibodies to SARS-CoV-19. Clin. Microbiol. Infect..

[15] Wald N.J., Watt H.C., Hackshaw A.K. (1999). Integrated screening for Down’s syndrome based on tests performed during the first and second trimesters. N. Engl. J. Med..

[16] Abu-Rustum R.S., Akolekar R., Sotiriadis A., Salomon L.J., Costa F.D.S., Wu Q., Frusca T., Bilardo C.M., Prefumo F., Poon L.C. (2020). ISUOG Consensus Statement on organization of routine and specialist obstetric ultrasound services in context of COVID-19. Ultrasound Obstet. Gynecol..

[17] Papageorghiou A.T., Ohuma E.O., Altman D.G., Todros T., Cheikh Ismail L., Lambert A., Jaffer Y.A., Bertino E., Gravett M.G., Purwar M. (2014). International Fetal and Newborn Growth Consortium for the 21st Century (INTERGROWTH-21st). International standards for fetal growth based on serial ultrasound measurements: The Fetal Growth Longitudinal Study of the INTERGROWTH-21st Project. Lancet.

[18] Cosma S., Borella F., Carosso A., Sciarrone A., Cusato J., Corcione S., Mengozzi G., Preti M., Katsaros D., Di Perri G. (2021). The “scar” of a pandemic: Cumulative incidence of COVID-19 during the first trimester of pregnancy. J. Med. Virol..

[19] Wang Q., Du Q., Guo B., Mu D., Lu X., Ma Q., Guo Y., Fang L., Zhang B., Zhang G. (2020). A Method to Prevent SARS-CoV-2 IgM False Positives in Gold Immunochromatography and Enzyme-Linked Immunosorbent Assays. J. Clin. Microbiol..

[20] Jiang C., Wang Y., Hu M., Wen L., Wen C., Wang Y., Zhu W., Tai S., Jiang Z., Xiao K. (2020). Antibody seroconversion in asymptomatic and symptomatic patients infected with severe acute respiratory syndrome coronavirus 2 (SARS-CoV-2). Clin. Transl. Immunol..

[21] Weatherbee B.A.T., Glover D.M., Zernicka-Goetz M. (2020). Expression of SARS-CoV-2 receptor ACE2 and the protease TMPRSS2 suggests susceptibility of the human embryo in the first trimester. Open Biol..

[22] Cosma S., Carosso A.R., Cusato J., Borella F., Carosso M., Bovetti M., Filippini C., D’Avolio A., Ghisetti V., Di Perri G. (2020). COVID-19 and first trimester spontaneous abortion: A case-control study of 225 pregnant patients. Am. J. Obstet Gynecol..

[23] La Cour Freiesleben N., Egerup P., Vauvert Römmelmayer Hviid K., Rosenbek Severinsen E., Kolte A.M., Westergaard D., Fich Olsen L., Prætorius L., Zedeler A., Hellerung Christiansen A.M. (2020). SARS-CoV-2 in first trimester pregnancy: A cohort study. Hum. Reprod..

[24] Pique-Regi R., Romero R., Tarca A.L., Luca F., Xu Y., Alazizi A., Leng Y., Hsu C.D., Gomez-Lopez N. (2020). Does the human placenta express the canonical cell entry mediators for SARS-CoV-2?. eLife.

[25] Rajagopalan S., Long E.O. (2018). Cell atlas reveals the landscape of early pregnancy. Nature.

[26] Faure-Bardon V., Isnard P., Roux N., Leruez-Ville M., Molina T., Bessieres B., Ville Y. (2020). Anatomical and timely assessment of protein expression of angiotensin-converting enzyme 2, SARS-CoV-2 specific receptor, in fetal and placental tissues: New insight for perinatal counseling. Ultrasound Obstet. Gynecol..

[27] Crovetto F., Crispi F., Llurba E., Figueras F., Gómez-Roig M.D., Gratacós E. (2020). Seroprevalence and presentation of SARS-CoV-2 in pregnancy. Lancet.

[28] Maru S., Patil U., Carroll-Bennett R., Baum A., Bohn-Hemmerdinger T., Ditchik A., Scanlon M.L., Krishnan P., Bogaert K., Woodbury C. (2020). Universal screening for SARS-CoV-2 infection among pregnant women at Elmhurst Hospital Center, Queens, New York. PLoS ONE.

[29] Menni C., Valdes A.M., Freidin M.B., Sudre C.H., Nguyen L.H., Drew D.A., Ganesh S., Varsavsky T., Cardoso M.J., El-Sayed Moustafa J.S. (2020). Real-time tracking of self-reported symptoms to predict potential COVID-19. Nat. Med..

[30] Ye Z., Wang L., Yang T., Chen L., Wang T., Chen L., Zhao L., Zhang S., Zheng Z., Luo L. (2019). Maternal Viral Infection and Risk of Fetal Congenital Heart Diseases: A Meta-Analysis of Observational Studies. J. Am. Heart Assoc..

[31] Iwasaki A., Yang Y. (2020). The potential danger of suboptimal antibody responses in COVID-19. Nat. Rev. Immunol..

